# Response of stem cells from different origins to biphasic calcium phosphate bioceramics

**DOI:** 10.1007/s00441-015-2116-9

**Published:** 2015-02-13

**Authors:** Sonja E. Lobo, Robert Glickman, Wagner N. da Silva, Treena L. Arinzeh, Irina Kerkis

**Affiliations:** 1 Department of Morphology and Genetics, Federal University of São Paulo, São Paulo, Brazil; 2 Department of Biomaterials & Biomimetics, College of Dentistry, New York University, New York, NY USA; 3 Department of Oral and Maxillofacial Surgery, College of Dentistry, New York University, New York, NY USA; 4 Department of Orthopaedic Surgery Prof. Matta Machado, Hospital da Baleia, Belo Horizonte, Minas Gerais Brazil; 5 Department of Biomedical Engineering, New Jersey Institute of Technology, Newark, NJ USA; 6 Department of Genetics, Butantan Institute, Avenida Vital Brasil, 1500, São Paulo, Brazil 05503-900; 7 Department of Surgery, School of Veterinary Medicine and Animal Science, University of São Paulo, Avenida Prof. Dr. Orlando Marques de Paiva, 87, São Paulo, Brazil 05508-270

**Keywords:** Hydroxyapatite, β-tricalcium phosphate, Mesenchymal stem cells, Adipose-derived stem cells, Dental pulp stem cells

## Abstract

Biphasic calcium phosphate (BCP) bioceramics have been successfully applied in a broad variety of presentation forms and with different ratios of hydroxyapatite (HA) and β-tricalcium phosphate (β-TCP). BCPs have been loaded with stem cells from different origins for bone tissue engineering purposes, but evidence of stem cell behavior on different compositions (various HA/β-TCP ratios) and physical features of BCPs is limited. We compared the adhesion, proliferation, viability and osteogenic potential of human mesenchymal stem cells (MSCs) on granular BCPs with equal HA/β-TCP ratio of diverse particle sizes and on porous blocks which had different chemical compositions. In addition, the osteogenic differentiation of MSCs was compared to adipose-derived (ADSC) and dental pulp (DPSC) stem cells, as well as to pre-osteoblasts on a particulate BCP. MSCs growing on granular BCPs demonstrated increased number as compared to MSCs growing on blocks. Cells proliferated to a greater extent on small granular BCPs, while large granular BCPs and blocks promoted cell differentiation. Surprisingly, the expression of genes involved in osteogenesis was upregulated in MSCs on bioceramics in basal medium which indicates that BCPs may have osteoinductive potential. This was confirmed with the upregulation of osteochondrogenic markers, at different time points, when stem cells from various tissues were grown on the BCP. This study demonstrates that BCPs, depending on their physical features and chemical composition, modulate stem cell behavior, and that stem cells from different origins are inherently distinct in their gene expression profile and can be triggered toward osteochondrogenic fate by BCPs.

## Introduction

**Fig. 1:**
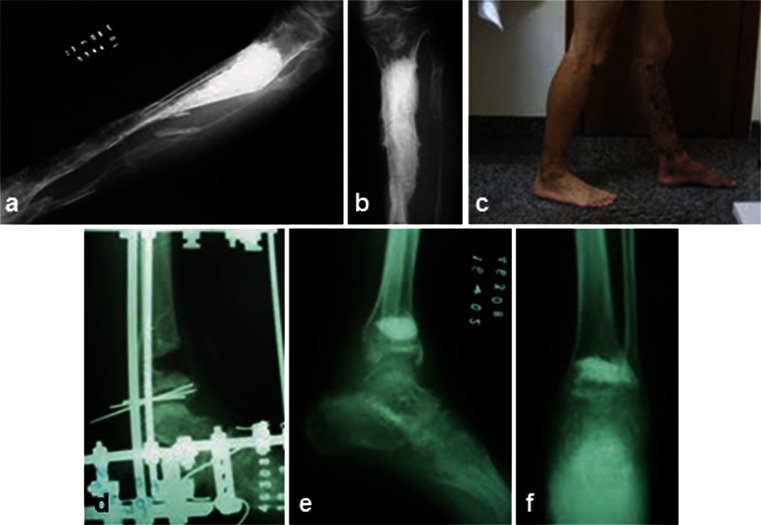
Reconstruction of tibial defects caused by tumors (**a**–**c**) and traumatic defects (**d**–**f**) using granules of BCP corresponding to 20-40 mesh. Postoperative x-rays of the tibia (**a**, **b**) and clinical aspect of the patient (**c**). Preoperative image of the bone defect (**d**) and its postoperative analysis in the lateral (**e**) and antero-posterior view (**f**) (provided by Dr. W.N. da Silva)

Successful reconstruction of critical size bone defects requires bone grafts or graft materials capable of restoring function by promoting new bone ingrowth and adequate biomechanical support. Biphasic calcium phosphate bioceramics (BCP) have been considered optimum bone graft substitutes due to their proven safety, osteoconductivity, bioactivity, biocompatibility, unlimited availability and their potential use as a scaffold for tissue engineering and drug delivery systems (Ohgushi and Caplan 1999; Le Nihouannen et al. 2007; Saldaña et al. 2009; Cheng et al. 2010; Lobo and Livingston Arinzeh 2010; Schumacher et al. 2010; Wang et al. 2010; Yuan et al. 2010). BCP scaffolds, composed of different ratios of hydroxyapatite (HA) and β-tricalcium phosphate (β-TCP), can have a variety of surface topographies, pore sizes and porosities (Arinzeh et al. 2005; Mastrogiacomo et al. 2006; Fellah et al. 2010) and have been widely used in several forms, such as granules or particulates, blocks, cements and custom-made implants. These biomaterials can also be fabricated as composites with natural and polymeric materials, thus fulfilling a broad range of clinical demands that include non-loading and load-bearing sites as well as the reconstruction of major bone defects (Ohgushi and Caplan 1999; Livingston et al. 2003; Marcacci et al. 2007; Matsushima et al. 2009; Saldaña et al. 2009; Schumacher et al. 2010; Teixeira et al. 2010; Weir and Xu 2010; Garrido et al. 2011; Fig. 1).

Biomaterials with different presentation forms have distinct clinical applications. For instance, granules and cements are normally used to fill bone cavities that result from bone cysts and tumors; granules are also used to restore areas where the need for faster bone formation surpasses the requirement for higher stability and mechanical strength. Alternatively, blocks, pre-shaped devices (such as wedges and cages) and custom-made implants (designed specifically for each patient) are usually for sites subjected to high loads and for aesthetic and accurate anatomical reconstruction immediately after the surgical procedure (de Oliveira et al. 2007; Garrido et al. 2011).

The diversity of tissue response and mechanical properties achieved in BCP scaffolds are a consequence of their chemical and physical structures. Some BCPs have been classified as osteoinductive due to their capacity to modify gene expression of human osteogenic and osteosarcoma cells and to heterotopically induce bone formation, in in vivo models, without the addition of bioactive molecules such as BMP (bone morphogenetic protein), mostly in large animal models (Rochet et al. 2003; Yuan et al. 2010; Barradas et al. 2011). Recent studies have shed light on molecular mechanisms that explain this intrinsic osteoinduction property which has been attributed primarily to the chemical composition and physical features, such as surface topography and size and shape of pores of the biomaterial (Ripamonti 1996; LeGeros 2008; Cheng et al. 2010; Yuan et al. 2010; Barradas et al. 2011; Shih et al. 2014; Xia et al. 2014).

Nanostructured BCPs, composed of 65 % HA/35 % ß-TCP (Osteosynt®; EINCO Biomaterial, Belo Horizonte, Minas Gerais, Brazil), which have interconnecting micro- and macropores (diameters <10 and >100 μm, respectively) (LeGeros et al. 2003; Lobo and Livingston Arinzeh 2010) and various forms, have been successfully used in orthopaedic and craniofacial applications (de Oliveira et al. 2007; Garrido et al. 2011; Fig. 1). However, their potential to be used as scaffolds in combination with stem cells for tissue engineering has not been investigated. Moreover, previous clinical studies have shown that the reconstruction of segmental bone defects that exceed 3 cm in length in long bones using these bioceramics may require more than one surgical procedure or a longer period of time to heal (Garrido et al. 2011). Thus, alternative therapeutic strategies are needed to reconstruct these challenging defects. The combination of stem cells with BCPs may be a viable approach.

Conversely, for bone tissue engineering applications, stem cells isolated from different tissues have been associated with a wide variety of biomaterials, including bioceramics, in order to enhance bone tissue formation (Laranjeira et al. 2013; Temple et al. 2014). Mesenchymal stem cells isolated from bone marrow (MSCs), adipose-derived stem cells (ADSC) and dental pulp stem cells (DPSC) are the most commonly studied and characterized adult stem cell types (Shi et al. 2001; Rosenbaum et al. 2008; Huang et al. 2009; Gronthos 2011; Utsunomiya et al. 2011; Kerkis and Caplan 2012; Harasymiak-Krzyzanowska et al. 2013). However, the literature is scarce on data that compares the response of different stem cell types cultured on the same biomaterial.

This study was performed based on the hypotheses that: (1) bioceramics presenting distinct chemical composition and physical features elicit differential stem cell response and may be osteoinductive, and that (2) stem cells isolated from various tissues behave differently when seeded on the same type of scaffold. This investigation aimed at analyzing the potential of bioceramics, which have been successfully applied in orthopaedic and craniofacial surgeries (de Oliveira et al. 2007; Garrido et al. 2011), to function as stem cell carriers and to induce their osteogenic differentiation, as well as to compare the potential of bioceramics that present the same chemical composition and physical structure to trigger osteogenic fate of adult stem cells from different origins. Therefore, the proliferation, viability and osteogenic differentiation of MSCs cultured on six different BCP scaffolds, divided into two groups, granules (G20-40, G40-60 and G60-80) and porous blocks (BCP1, BCP2 and BCP3), were evaluated. The granules had the same HA/TCP ratio but varied in physical structure, i.e., granule size. The blocks differed in composition. Cell proliferation and viability were measured using two different assays, DNA content and metabolic activity, over time. The osteogenic differentiation was evaluated by gene expression and alkaline phosphatase activity over time. Moreover, the determination of osteogenic cell fate of MSC, ADSC and DPSC on a granular form of BCP (G20-40) was compared to pre-osteoblasts. Cell adhesion was analyzed using scanning electron microscopy and the expression of genes responsible for the determination of stem cell fate was investigated using RT-PCR.

## Materials and methods

### Materials

Nanostructured micro- and macroporous biphasic calcium phosphate (BCP)-based bioceramics were provided by EINCO Biomaterial in the form of granules and blocks. The granules had different particle sizes and were irregular in shape but had the same chemical composition: 65/35 (wt%/wt%) HA/ß-TCP. They were named: G20-40 (corresponding to granules of 20–40 mesh, with largest diameter of up to 1.5 mm); G40-60 (granules of 40–60 mesh, where the largest diameter corresponded to 1 mm); and G60-80 (corresponding to particles of 60–80 mesh, which had an average of 0.7 mm at the largest diameter). The blocks, 5 mm in diameter and 2 mm in thickness, had different chemical compositions: BCP1 was composed of 65/35 % HA/ß-TCP, i.e., the same composition of the granules, BCP2 was a composite of HA and poly(methyl 2-methylpropenoate), and BCP3 was composed of 10 %/90 % HA/β-TCP ratio (Table 1). The chemical composition was verified by XRD (data not shown). The surface topographies were assessed qualitatively by scanning electron microscopy (SEM) viewing of the scaffolds’ surface. For the comparative analysis of osteogenic differentiation of stem cells from distinct origins, the granules G20-40 were used.

**Table 1:** Chemical composition, physical forms and porosity of the six different BCP scaffolds

Samples		Chemical composition (HA/β-TCP ratio)	Physical form	Porosity	
				Micro	Macro
Bioceramics	G20-40	65%/35%	Granules	***	***
	G40-60	65%/35%	Granules	***	**
	G60-80	65%/35%	Granules	***	*
	BCP1	65%/35%	Blocks	***	**
	BCP2	100% HA/polymer	Blocks	***	***
	BCP3	10%/90%	Blocks	***	**

The BCPs were granules (G20-40, G40-60 and G60-80) and blocks (BCP1, BCP2 and BCP3). Granules had the same microporosity (<10 μm) but different particle sizes and macroporosity (>100 μm) (observed by SEM). The granules and BCP1 had the same chemical composition but different physical features. Blocks had different compositions and surface topographies. For porosity, a semi-quantitative grading system varying from 3 to 1 asterisks was used for high to low porosity, respectively

### Cell culture

Human mesenchymal stem cells (MSCs) were isolated from whole bone marrow from male donors, 18–30 years old (Lonza, USA), as previously reported (Briggs et al. 2009), and cultured in control media composed of Dulbecco’s Modified Eagle’s Medium low glucose (Gibco; Invitrogen, Carlsbad, CA, USA), supplemented with 10 % fetal bovine serum (HyClone; Thermo Fisher Scientific, USA) and 100 units/ml antibiotic-antimycotic (Invitrogen). Adipose-derived stem cells (ADSC; Poietics Human Adipose-Derived Stem Cells) were purchased from Lonza (Walkersville, MD, USA) and grown in ADSC Growth Medium (Bulletkit; Lonza). Dental pulp stem cells (DPSC) were isolated from third molars extracted at the Department of Oral Surgery at New York University College of Dentistry (reviewed and approved by the Committee of Ethics), as previously reported (Kerkis et al. 2006). Briefly, after extraction, teeth were cleaned with povidine, and odontosection was performed at the cemento-enamel level using a low rotation saw under profusion irrigation with saline solution. After exposure of the pulp chamber, pulp tissue was removed from the crown and roots, under sterile conditions, and placed in Petri dishes with DMEM/F12 media (Gibco; Invitrogen) supplemented with 15 % FBS (HyClone®; Thermo Fisher Scientific), 100 units/ml of penicillin/streptomycin (Sigma-Aldrich, St. Louis, MO, USA), 2 mM L-glutamine (Gibco; Invitrogen) and 2 mM non-essential amino acids (Gibco, Invitrogen). The pulp was incubated at 37 °C, 5 % CO_2_, for separation of stem cells from the extracellular matrix through explant. Cells have been characterized elsewhere with regard to their multipotency and cell membrane markers and shown to be stem cells. Human pre-osteoblasts (MG-63; Lonza) were cultured in Alpha-MEM (Minimum Essential Medium Alpha; Gibco, Invitrogen), supplemented with 10 % FBS (HyClone; Thermo Fisher Scientific) and 100 units/ml of penicillin/streptomycin (Sigma-Aldrich).

Cells were expanded in control medium (basal medium) until passage 3 and seeded onto the BCP scaffolds at 3 × 10^4^cells/cm^2^ in polypropylene plates. Cells on scaffolds were grown in control and osteogenic media (OS), comprised of control media supplemented with 100 nM of dexamethasone, 0.05 mM of ascorbic acid and 10 mM of β-glycerophosphate. As control groups, cells were seeded directly on tissue culture polystyrene plates (TCP) and cultured in both control (C) and osteoinductive medium (OS), without scaffolds. In order to avoid eventual influences on cell growth, viability, adhesion and/or differentiation provided by the growth media, the osteogenic media was used in the respective groups from the time of cell seeding (i.e., control media was not initially applied and later changed to OS media). Media was changed every 2–3 days and the cells were kept in a 5 % CO_2_ incubator at 37 °C.

### Scanning electron microscopy

The physical features (presence of pores and surface topographies) of the BCPs as well as stem cells and pre-osteoblast morphologies and adhesion to the scaffolds were observed using SEM (Hitachi S-3500 N LV-SEM, Singapore). The constructs (stem cells on scaffolds) were washed twice with PBS and fixed with Karnovsky solution (Karnovsky Fixative, Electron Microscopy Sciences, Hatfield, PA, USA). The samples were then subjected to serial dehydration with 10, 30, 50, 70, 80, 90, and 100 % ethanol, dried overnight with 1,1,2-triclorofluorethane and gold-coated. Bioceramics without stem cells were directly coated with gold. Two samples of each scaffold were analyzed and the constructs were evaluated after 4 h, and 1, 7 and 14 days of culture.

### Cell proliferation

Cell number was quantified using Quanti-iT PicoGreen dsDNA Assay Kit (Molecular Probes, Invitrogen, USA). Briefly, the cells were lysed using 0.1 % Triton X-100 for 30 min and incubated at room temperature for 5 min with the fluorochrome PicoGreen, which stains the nucleic acid by selective binding to the double-stranded DNA. The plates were read on the spectrophotometer FLX800 microplate reader using KC Junior software at wavelength of 480 nm and emission corresponding to 520 nm. Fours samples per group per time point were studied; the assay was repeated twice and the analysis was performed at days 4, 7, 11 and 14.

### Cell viability

The cell viability was quantified using the XTT assay (XTT Cell Viability Assay Kit; Biotium, Hayward, CA, USA), which measures the activity of the mitochondrial enzymes, according to the manufacturer’s protocol. The cell viability was determined using a standard curve correlating absorbance values to standards of known cell numbers. The absorbance was measured with a spectrophotometer at wavelength of 450 nm. Four samples per group per time point were studied and the assays were repeated twice. Samples were analyzed at 4, 11 and 14 days of culture.

### qRT-PCR

The total RNA was isolated using Qiagen RNAeasy Kit (Qiagen, CA, USA) and the DNA was excluded using RNase Free DNase Set. Quantitative reverse transcriptase-polymerase chain reaction (qRT-PCR) was performed using One Step QuantiTect SYBR Green RT-PCR Kit (Qiagen), according to the manufacturer’s instructions. Specific oligonucleotide primers for bone sialoprotein (integrin-binding sialoprotein, IBSP; QT00093709); osteopontin (secreted phosphoprotein 1, SPP; QT01008798); RUNX2 (runt-related transcription factor 2; QT00020517); SOX9 (SRY [sex determining region Y]-box 9; QT00001498); PPARγ (peroxisome proliferator-activated receptor; QT00029841); SOX2 (SRY-box 2; QT00237601) were analyzed and their relative gene expression was normalized to the relative expression of the housekeeping gene RPLP0 (ribosomal protein P0; QT01839887) or GAPDH (glyceraldehyde-3-phosphate dehydrogenase; QT01192646). Samples were analyzed at days 1, 3, 7 and/or 14, assayed in triplicate for each time point, and repeated twice. The reverse transcription was performed for 30 min at 50 °C, the PCR activation for 15 min at 95 °C, and the amplification reactions were carried out through 40 cycles (15 s denaturation at 94 °C, 30 s of annealing at 55 °C and 30 s of extension at 72 °C) using the MX4000 detection system (Stratagene, USA).

### Alkaline phosphatase activity

The alkaline phosphatase activity was measured using the p-Nitrophenyl phosphate assay (Sigma-Aldrich, USA) according to the manufacturer’s protocol. The absorbance was measured at 405 nm using a spectrophotometer (Molecular Devices, USA). Four samples per group per time point were studied and the assay was repeated twice. Alkaline phosphatase activity was normalized to cell number, i.e., data from the proliferation analysis (Quanti-iT PicoGreen dsDNA Assay). The analyses were performed at days 7, 11 and 14.

### Statistical analysis

Statistical analysis was performed using Bonferroni multiple-significance test where comparisons for each one and between the variables (time points, cell culture media, with and without biomaterials and differences among biomaterials) were performed (*p * < 0.05).

## Results

**Fig. 2:**
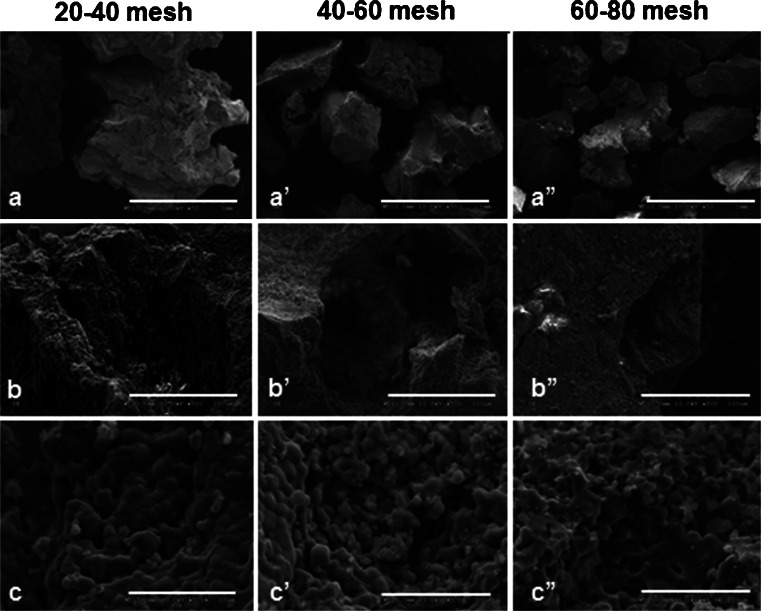
SEM images of the granules with different particle sizes (20–40 mesh, 40–60 mesh, and 60–80 mesh). The shape and size of the granules (**a**, **a’**, **a”**), the size and presence of macropores (**b**, **b’**, **b”**) and the micropores (**c**, **c’**, **c”**) can be observed. *Bars* (**a**, **a’**, **a”**) 1 mm, (**b**, **b’**, **b”**) 100 µm, (**c**, **c’**, **c”**)  10 µm

**Fig. 3:**
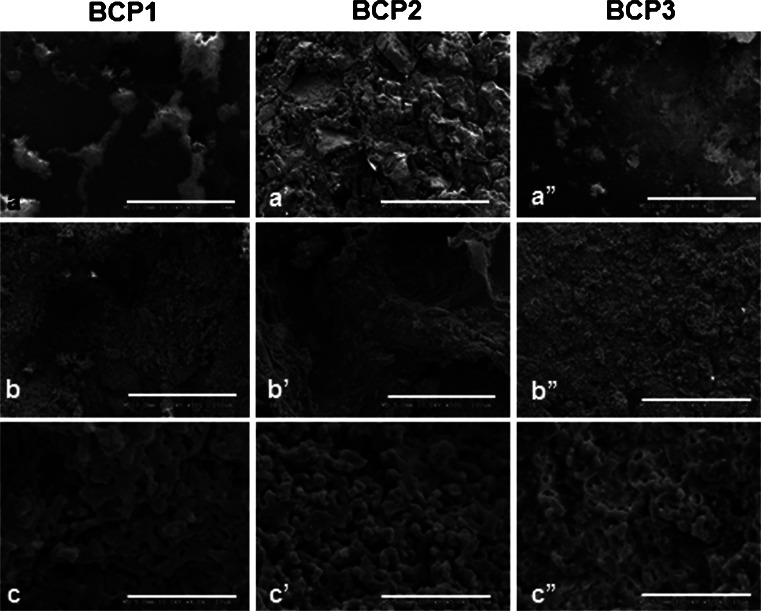
SEM images of the blocks (BCP1, BCP2 and BCP3) showing distinct surface topographies (**a**, **a’**, **a”**). Variation in the size and amount of macropores (**b**, **b’**, **b”**) were seen whereas the presence of micropores (**c**, **c’**, **c”**) were observed in all of the scaffolds. Bars (**a**, **a’**, **a”**) 1 mm, (**b**, **b’**, **b”**) 100 µm, (**c**, **c’**, **c”**) 10 µm

**Fig. 4:**
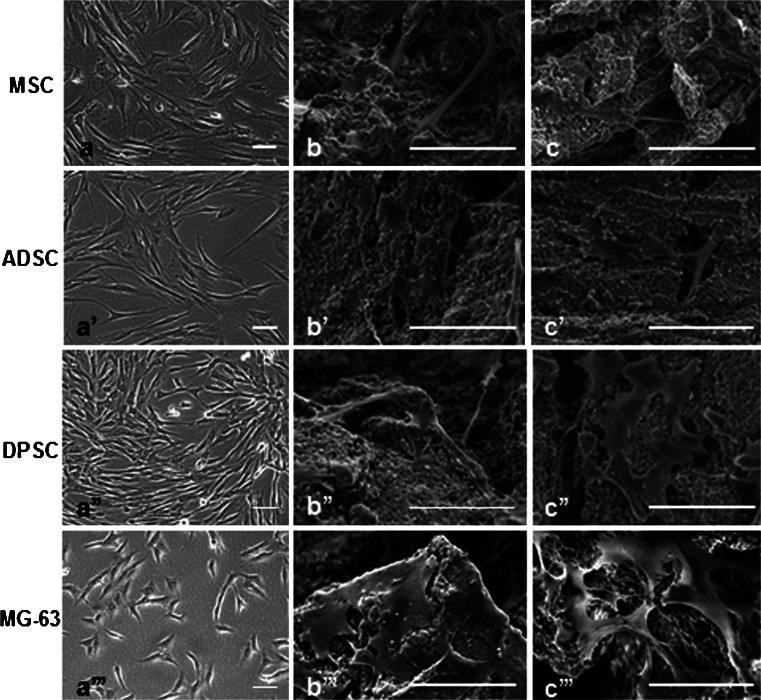
Morphological aspects of stem cells and pre-osteoblasts on tissue culture plate (**a**, **a’**, **a”**, **a”’**) and on bioceramics G20-40 (**b**, **b’**, **b”**, **b”’** and **c**, **c’**, **c”**, **c”’**), in control media, at day 7. Under light microscopy, pre-osteoblasts (*MG-63*) had round-shaped morphology compared to stem cells (**a**, **a’**, **a”**, **a”’**). When seeded on the bioceramics, eventual distinct morphological features of the cells are not distinguishable (**b**, **b’**, **b”**, **b”’**, **c**, **c’**, **c”**, **c”’**). *MSC* mesenchymal stem cells, *ADSC* adipose-derived stem cells, *DPSC* dental pulp stem cells. *Scale bars* (SEM) 50 μm

**Fig. 5:**
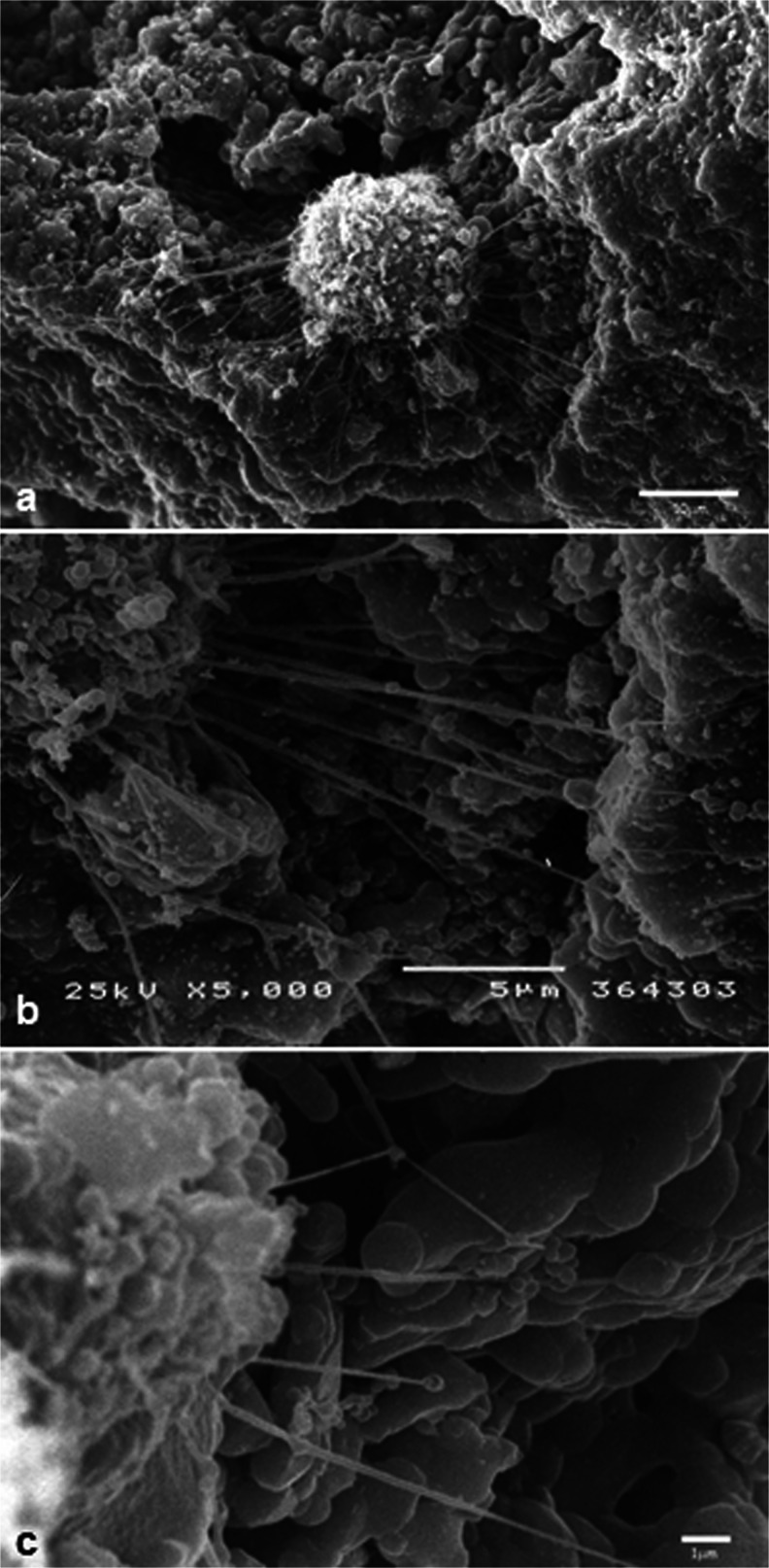
Initial adhesion of a dental pulp stem cell, 4 h after seeding on granules G20-40, mediated by the emission of filopodia-like structures. *Scale bars* (**a**) 10 μm, (**b**-) 5 μm, (**c**) 1 μm

All scaffolds had nanostructured surfaces with similar micropores (<10 μm) as observed by SEM. The macropores (>100 μm) as well as the surface topographies appeared to vary among the six scaffolds. Macropores were observed in all the scaffolds but to a lesser extent in the G60-80 group. Among the blocks, the group BCP2 had the most irregular surface and appeared to have more macropores (Figs. 2 and 3). Cells were observed to adhere to the surface of all the scaffolds and to penetrate their porous structure. Stem cells from various tissues and pre-osteoblasts had slightly different morphologies when cultured directly on polystyrene plates (Fig. 4). Dental pulp stem cells were found to extend multiple filopodia-like protrusions within hours after seeding which allowed them to adhere to the surfaces of the bioceramics (Fig. 5). Thereafter, cells spread on the surface and inside the pores of the BCP scaffolds (Fig. 4).

**Fig. 6:**
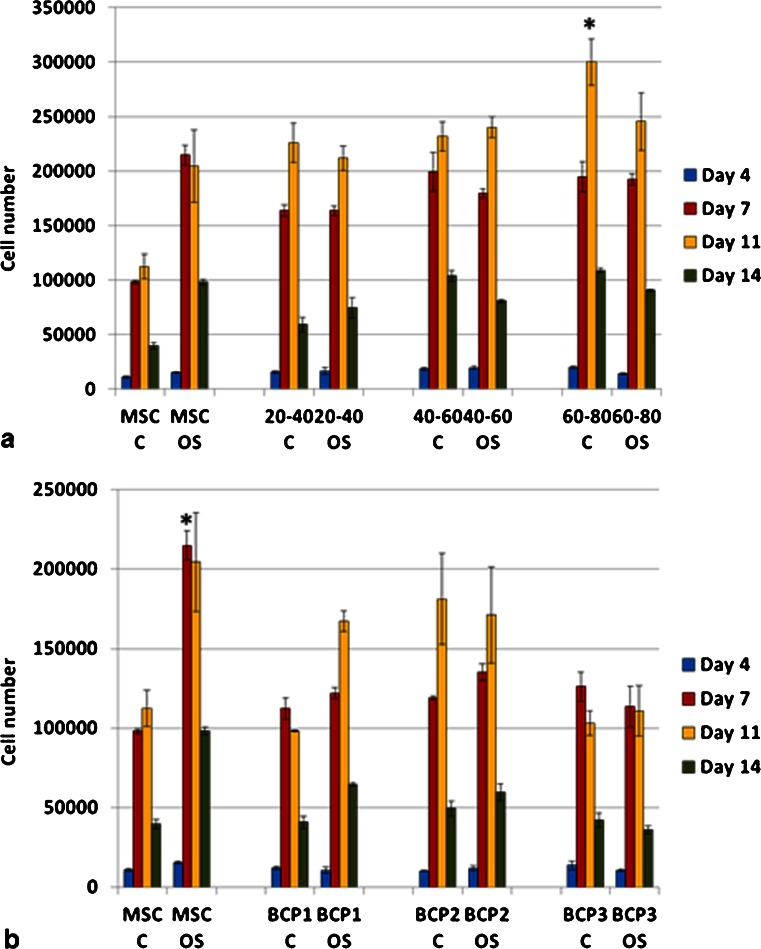
Mesenchymal stem cell proliferation, evaluated by Pico-Green assay, on granules (**a**) and blocks (**b**). Higher cell number was determined on granules as compared to all the blocks. Among the granules, the highest cell number among all groups was for the smallest particles (60–80 mesh) in control media (*p < 0.05). Among the blocks, higher cell number was determined for BCP2 but was not statistically significant. *C* control media; *OS* osteogenic media (*p < 0.05). Values are mean ± SE

**Fig. 7:**
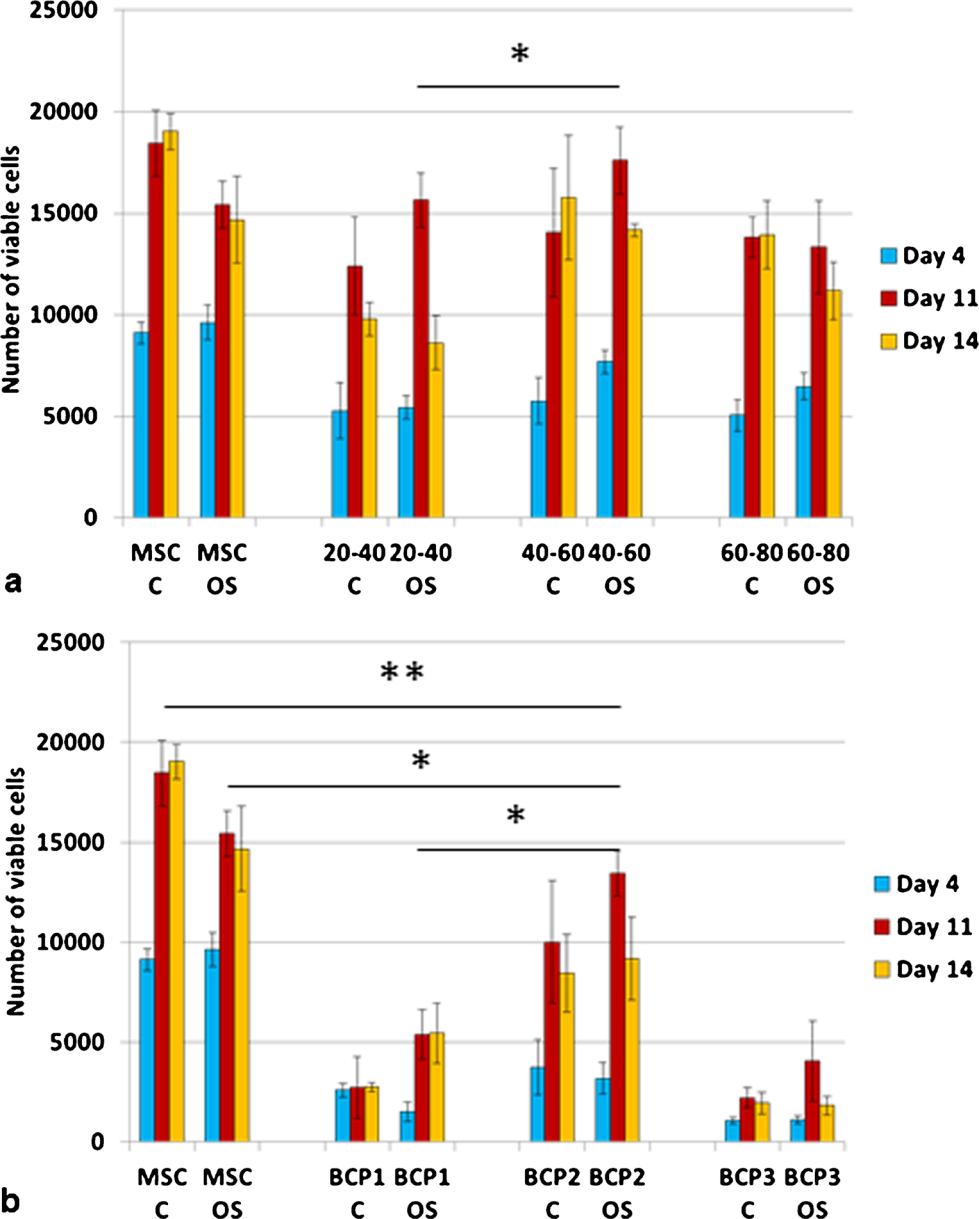
Mesenchymal stem cell viability analyzed by XTT assay showed that the granules (**a**), had higher viable cells as compared to the blocks (**b**) (*p* < 0.05). The BCP2 had the highest number of viable cells among the blocks (*p < 0.05; ** *p* < 0.01). Values are mean ± SE

Cell number was higher on granules as compared to blocks (Fig. 6). Almost all groups showed the highest cell number at day 11 of culture. The cells cultured directly on polystyrene plates showed higher number in osteogenic media as compared to the control media (*p* < 0.05). However, cells cultured on the BCPs did not show statistically significant differences between both media (control and osteogenic). The group G60-80 (granules with the smallest particle size, therefore presenting higher surface area for cell adhesion) in control media showed the highest cell number at day 11 (*p* < 0.05) (Fig. 6a). Among the blocks, cell number increased in the group BCP2, although it was not statistically significant and was lower than the control group in osteogenic media (MSC OS) (Fig. 6b). Due to the fact that no significant differences in cell proliferation were observed between days 7 and 11 and that the latest (day 11) presented the highest cell number in some experimental groups, day 7 was not included in further assays. The groups G40-60 and BCP2 had the highest number of viable cells amongst all bioceramic groups and G40-60 was equivalent to the control group (Fig. 7).

**Fig. 8:**
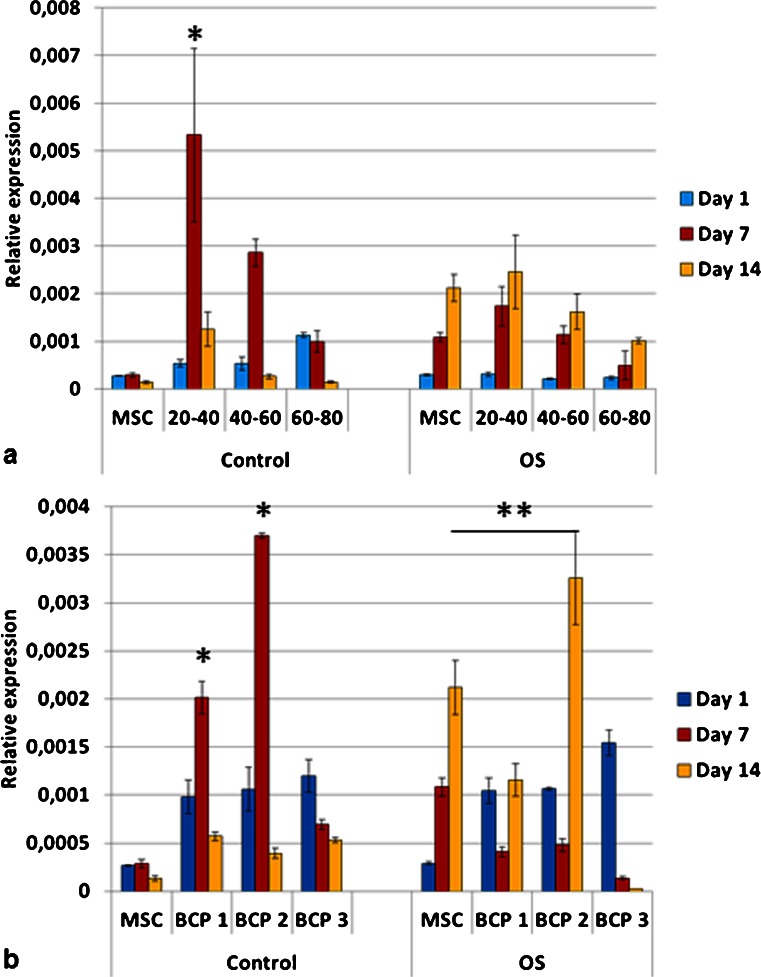
Relative expression of bone sialoprotein (BSP) for mesenchymal stem cells on the granules (**a**) and blocks (**b**), where the values were normalized to the housekeeping gene (RPLP0). Cells cultured in OS media either on tissue culture polystyrene or on granules had increased BSP expression from day 1 to day 14 (*p* < 0.05). Cells grown on granules of 20–40 mesh in control media, at day 7, showed the highest level of mRNA for BSP amongst all groups, including other granules and all blocks (*p < 0.05). Cells grown on granules (20–40 mesh) and blocks (BCP1 and BCP2) had higher levels of mRNA for BSP in control media in comparison to OS media at day 7 (*p < 0.05). At day 14, statistical differences were detected between BCP2 OS and MSC OS (**p < 0.05). Among blocks, BCP2 at day 7 in control media had the highest expression of BSP (*p < 0.05). *OS* osteogenic media. Values are mean ± SE

The expression of bone sialoprotein (BSP) for cells grown on TCP (tissue culture plate) and on granules in OS medium increased over time (Fig. 8a). Interestingly, cells cultured on BCP1 and BCP2 in control medium had an upregulation of BSP as compared to OS medium at day 7. This increased expression was also observed for granules 20–40 mesh (G20-40) and 40–60 mesh (G40-60) at day 7 in control medium as compared to OS medium (*p* < 0.05). BSP expression decreased on BCP3 group in both media over time (Fig. 8b). High level of BSP was detected for the granules of 20–40 mesh in control media at day 7 (Fig. 8).

**Fig. 9:**
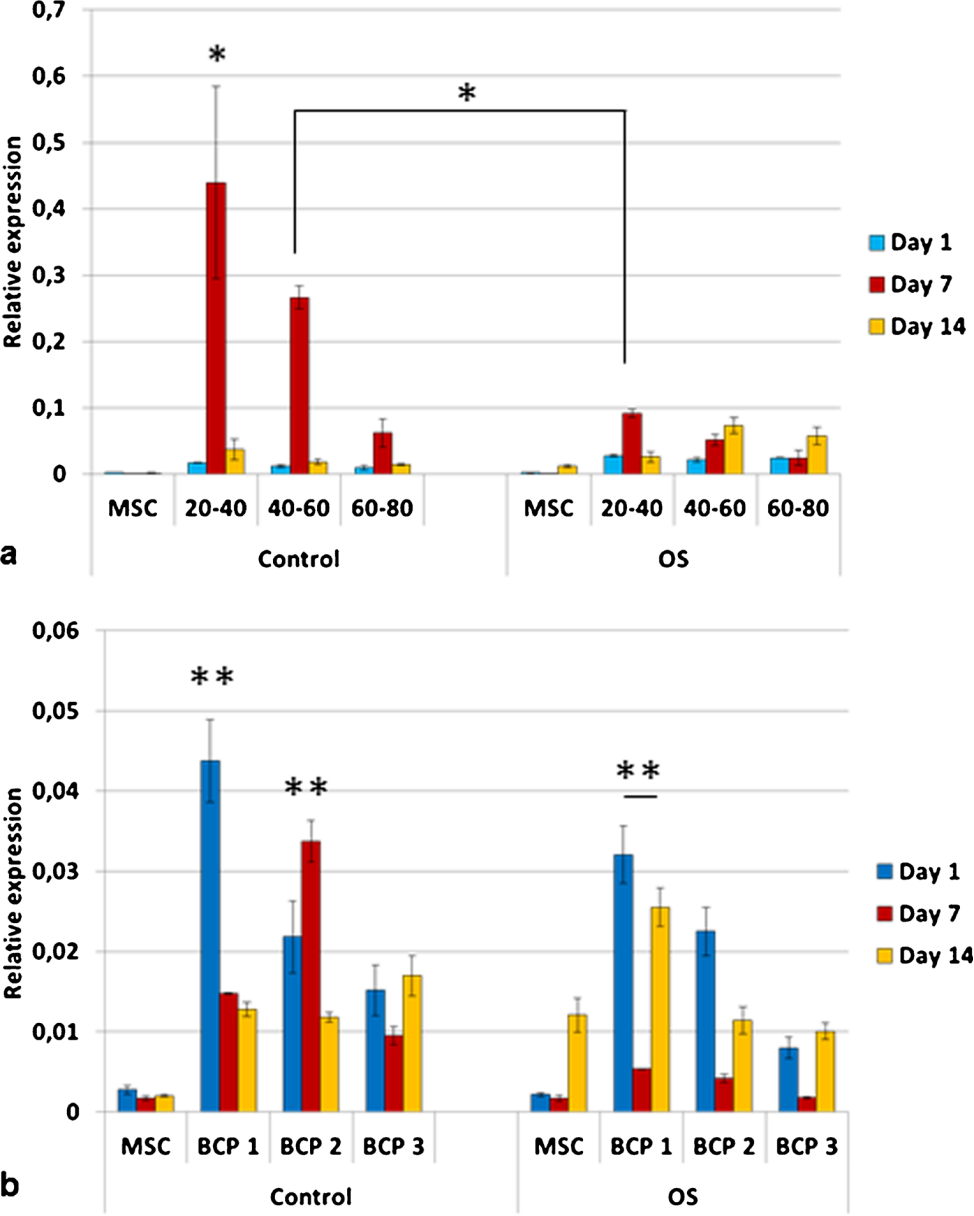
Relative expression of osteopontin (OPN) for mesenchymal stem cells on granules (**a**) and blocks (**b**) (values were normalized to the housekeeping gene RPLP0). The mRNA levels were higher for cells cultured on the granules for all groups and time points compared to the blocks (*p < 0.05). Cells cultured on both G20-40 and G40-60 granules in control medium had higher levels of osteopontin expression as compared to cells cultured on the same biomaterials but in OS medium at day 7 (*p < 0.05). Cells on G20–40 had higher expression of osteopontin than all groups (*p < 0.05). Cells on BCP1 in control medium at day 1 and BCP2 in control medium at day 7 had the highest osteopontin expression among blocks (**p < 0.05). Cells on BCP1 in OS medium had higher osteopontin expression as compared to MSCs on tissue culture polystyrene in OS medium at all time points (**p < 0.05). *OS* osteogenic medium. Values are mean ± SE

The expression of osteopontin (OPN) was higher for cells cultured on the bioceramics (granules and blocks) in both media than on TCP (control groups, i.e., MSC C and MSC OS) (Fig. 9). Exception was observed for cells on blocks BCP3 OS at days 7 and 14 and BCP2 OS at day 14, which had equivalent OPN gene expression compared to the group MSC OS. Cells on granules G20-40 in control medium presented the highest OPN expression at day 7 amongst all groups (Fig. 9a). Within the blocks, upregulation of OPN was observed for cells grown on BCP1 (days 1, 7 and 14) and BCP2 (day 7) in control medium as compared to OS medium (Fig. 9b).

**Fig. 10:**
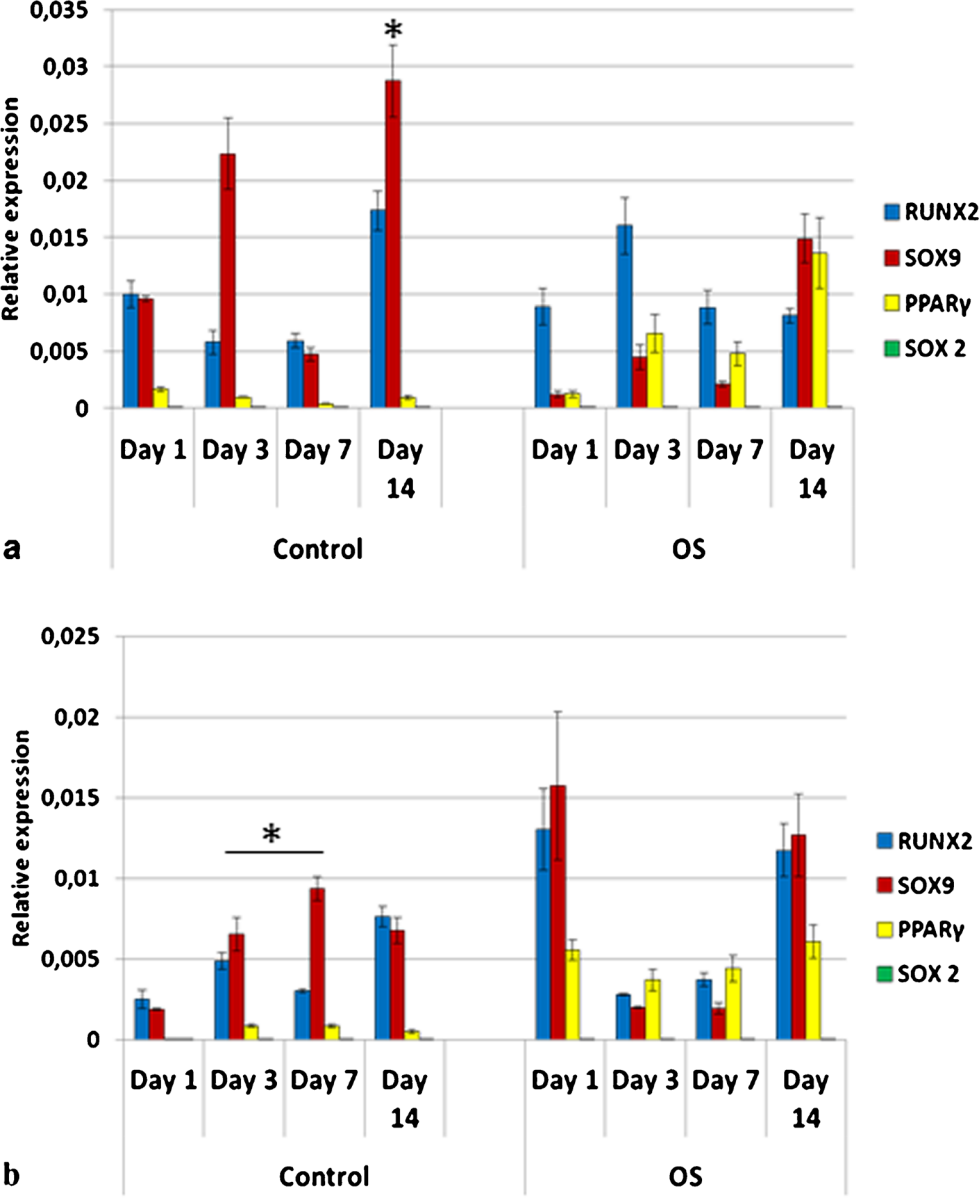
Gene expression profile of mesenchymal stem cells (MSC) seeded on tissue culture plate (TCP) (**a**) and on biphasic calcium phosphate bioceramics (BCP) (**b**), in control and OS media. Predominance of RUNX2 and SOX9 co-expression was observed mainly in control media in both surfaces (TCP and BCP), as well as on BCP in OS medium, indicating osteochondrogenic differentiation. PPARγ was upregulated when OS medium was used. *OS* osteoinductive medium (*p < 0.05). Values were normalized to the housekeeping gene GAPDH. Values are mean ± SE

**Fig. 11:**
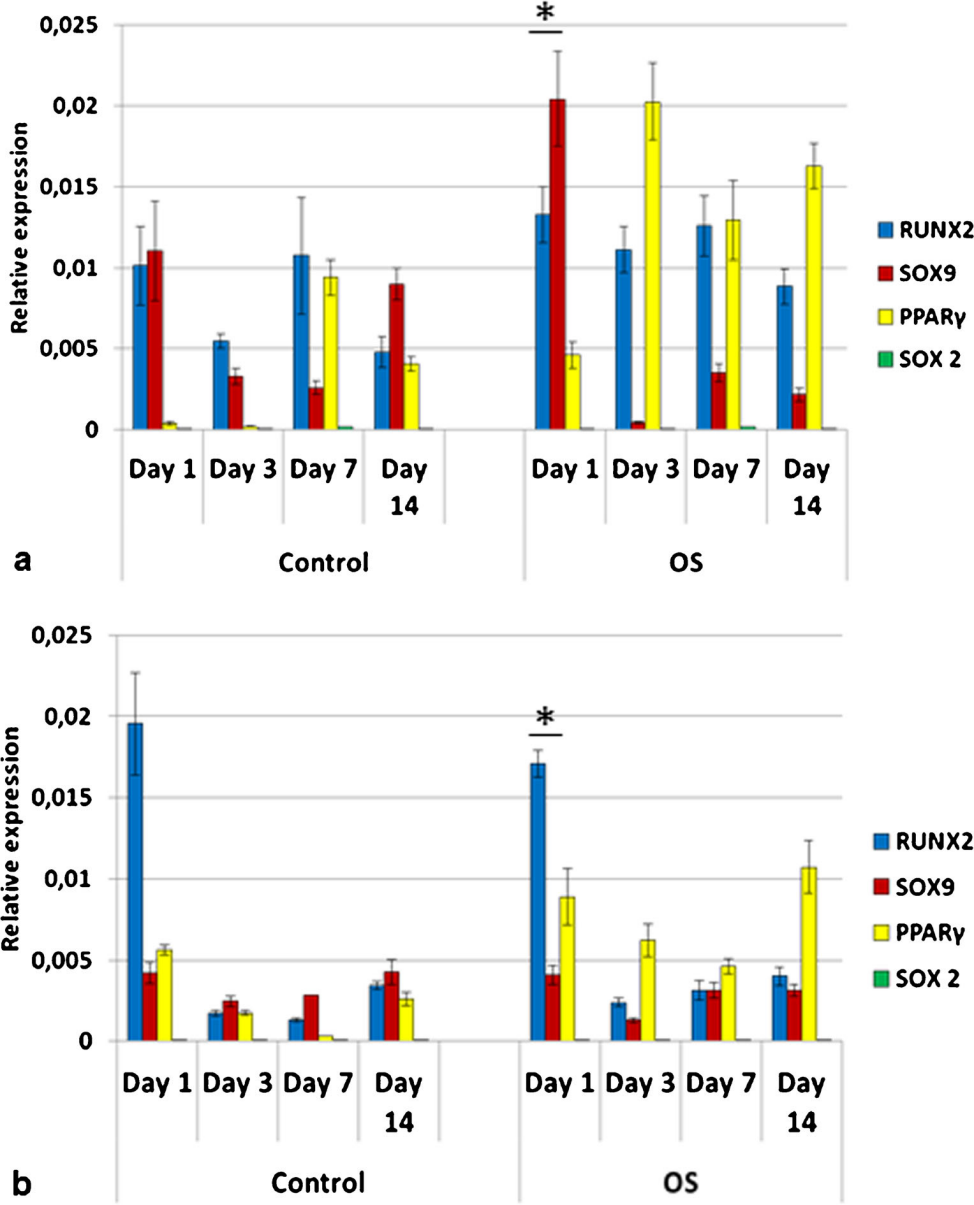
Gene expression profile of adipose derived stem cells (ADSC) seeded on tissue culture plate (TCP) (**a**) and on biphasic calcium phosphate bioceramics (BCP) (**b**), in both control and OS media. Co-expression of RUNX2 and SOX9 was observed in all experimental conditions. However, significant upregulation of PPARγ was observed when cells were cultured in OS media, mostly on TCP, although its expression was also detected in control media. *OS* osteoinductive medium (*p < 0.05). Values were normalized to the housekeeping gene GAPDH. Values are mean ± SE

**Fig. 12:**
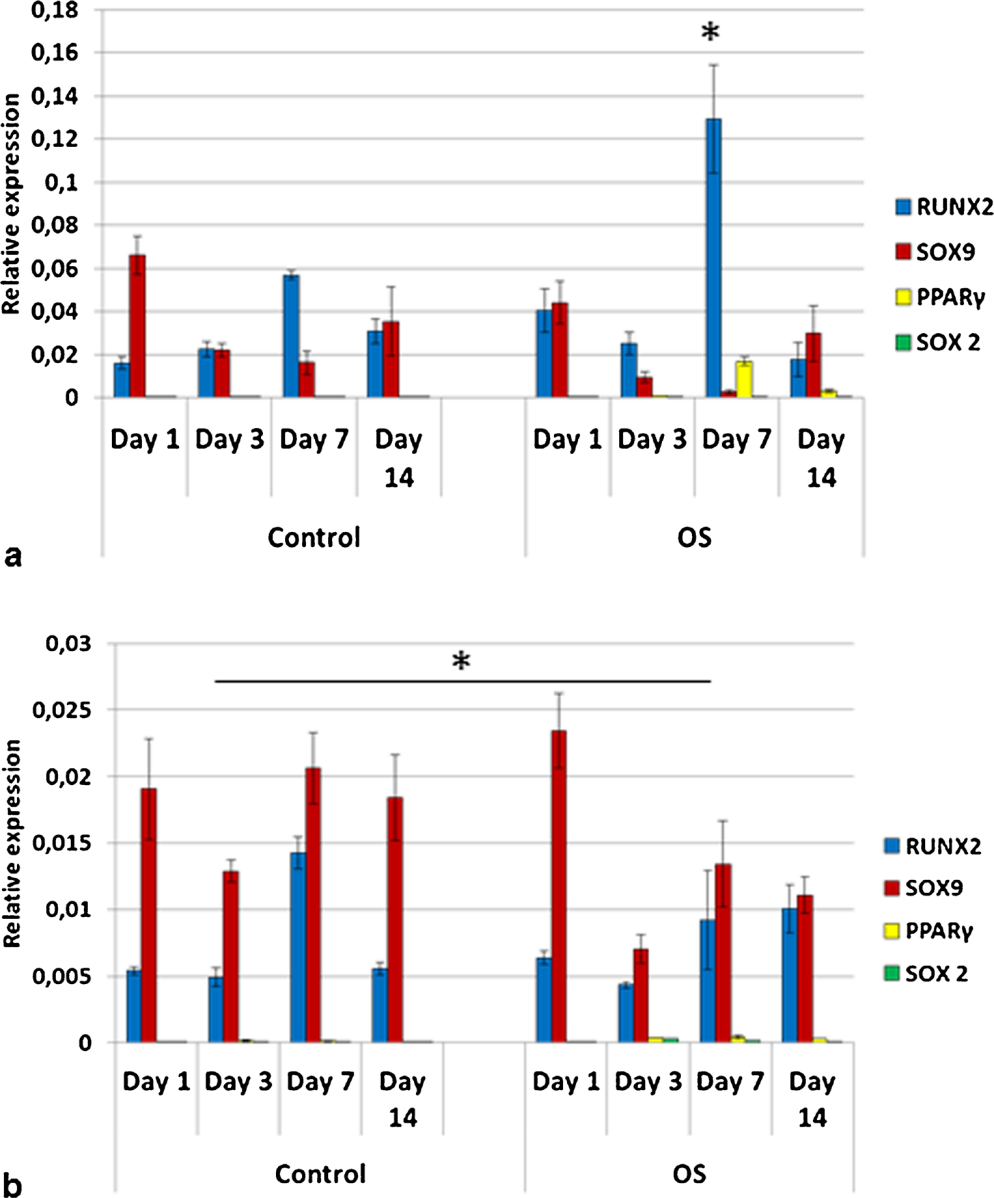
Gene expression profile of dental pulp stem cells (DPSC) seeded on tissue culture plate (TCP) (**a**) and on biphasic calcium phosphate bioceramic (BCP) (**b**), in both control and OS media. Cells did not express PPARγ, except in OS medium on TCP (**a**). When cultured on BCP in both media, DPSC showed upregulation of SOX9 over RUNX2 at all time points (**b**). *OS* osteoinductive medium (*p < 0.05). Values were normalized to the housekeeping gene GAPDH. Values are mean ± SE

Due to the fact that the G20-40 group showed high BSP and OPN gene expression in control medium, it was used for the comparison of genes expressed at early stages of differentiation by stem cells from different origins as well as pre-osteoblasts. MSC, ADSC and DPSC had distinct profiles of mRNA expression for RUNX2, SOX9 and PPARγ when cultured on TCP and in control (C) media (Figs. 10a, 11a and 12a).

MSC C (control media) on TCP had predominant co-expression of RUNX2 and SOX9 and very low levels of PPARγ, at all time points (Fig. 10a). MSC OS (osteogenic media) on TCP had increased expression of PPARγ over time, with a peak on day 14. Furthermore, levels of RUNX-2 also increased from days 1 to 14, whereas SOX9 was concurrently downregulated. SOX9 had the highest expression for MSC C on TCP on day 14 (Fig. 10a). MSC C on BCP (G20-40) significantly co-expressed RUNX2 and SOX9 throughout the study, and very low levels of PPARγ were detected (Fig. 10b). On day 7, SOX9 expression for MSC C on BCP was higher than those for MSC C on TCP (*p* < 0.05). The levels of RUNX2 and SOX9 for MSC C on BCP on day 3 and of SOX9 on day 7, in the same conditions, were higher than those for MSC OS on BCP (Fig. 10b). MSC OS on BCP showed marked co-expression of RUNX2 and SOX9 on day 1, which was higher than that for all other groups at the same time point. Conversely, levels of PPARγ were higher on MSC OS on BCP in comparison to MSC C on BCP (Fig. 10b).

ADSC C on TCP expressed RUNX2 and SOX9 on days 1 and 3 when PPARγ was not detected. However, on days 7 and 14, the expression of PPARγ was significantly increased for ADSC C (Fig. 11a). ADSC OS on TCP expressed PPARγ at all time points, differently from ADSC C on TCP (Fig. 11). These cells showed simultaneous upregulation of RUNX2 and SOX9 on day 1 in OS media but, at the later time points, SOX9 was downregulated whereas the expressions of RUNX2 and PPARγ remained high (Fig. 11b). ADSC C on BCP showed simultaneous expression of RUNX2, SOX9, and PPARγ at all time points, although on day 1, RUNX2 was significantly upregulated (Fig. 11b). The levels of PPARγ were lower for ADSC C on BCP compared to those of all other groups; an exception was observed on day 1 for ADSC C on BCP, which expressed more PPARγ compared to ADSC C on TCP. PPARγ was significantly downregulated for ADSC C on BCP in comparison to ADSC C on TCP, on days 3, 7, and 14. On the other hand, ADSC OS on BCP showed increased expression of PPARγ compared to ADSC C on BCP for all time points (Fig. 11b). These values were, however, lower than those for ADSC OS on TCP. The expression of RUNX2 and SOX9 were similar for ADSC C on BCP and for ADSC OS on BCP. On day 1, RUNX2 for ADSC C on BCP was higher than that for ADSC OS on BCP, although this difference was not statistically significant (Fig. 11).

On TCP, DPSC did not express PPARγ in C media, at any time point but co-expressed RUNX2 and SOX9 (Fig. 12a). Both DPSC C on TCP and DPSC OS on TCP expressed mostly RUNX2 and SOX9 (Fig. 12a), except for DPSC OS on TCP, in which case low levels of PPARγ were detected on day 7 (Fig. 12a). The absence and very low levels of PPARγ observed with DPSC is in contrast with the relatively very high levels detected for ADSC. The highest gene expression shown by DPSC was for RUNX2, at day 7, in OS media, on TCP. DPSC C and DPSC OS, both on BCP, expressed RUNX2 and SOX9 only (Fig. 12b). In addition, for DPSC on BCP, in both C and OS media, the SOX9 expression was higher than RUNX2 at all time points except on day 14, when DPSC OS on BCP had equivalent values of RUNX2 and SOX9 (Fig. 12b). DPSC C on BCP elicited higher SOX9 expression than DPSC OS on BCP on days 3, 7, and 14. On days 3 and 7, SOX9 was higher for DPSC C on BCP than for DPSC OS on BCP (Fig. 12b).

**Fig. 13:**
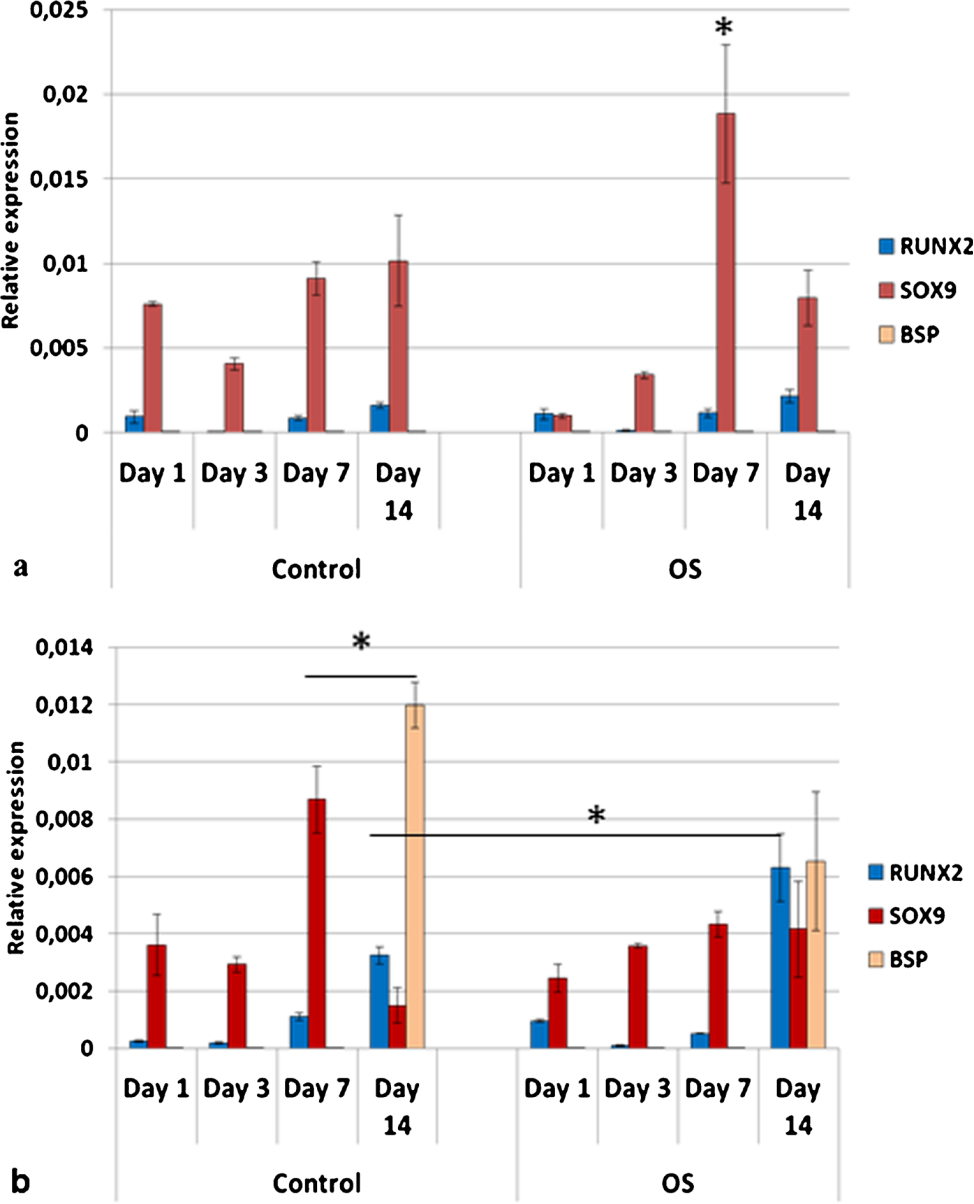
Gene expression profile of pre-osteoblasts (MG-63) seeded on tissue culture plate (TCP) (**a**) and on biphasic calcium phosphate bioceramics (BCP) (**b**), in both control and OS media. Significant expression of SOX9 was observed in all experimental conditions. MG-63 expressed bone sialoprotein only on BCP (**b**) but not on TCP (**a**). On BCP, BSP levels were higher in control as opposed to OS media (**b**). RUNX2 was detected at later time points (days 7 and 14) and was upregulated on BCP as compared to TCP. RUNX2 expression was higher on BCP OS than on BCP control, on day 14. *OS* osteoinductive medium (*p < 0.05). Values were normalized to the housekeeping gene GAPDH. Values are mean ± SE

Pre-osteoblasts (MG-63) in C and OS media, on TCP, remarkably expressed SOX9 at all time points (Fig. 13a). On day 1, the levels of RUNX2 and SOX9 for MG-63 OS on TCP were equivalent but, at later time points, SOX9 was upregulated, being significantly higher than RUNX2. No expression of bone sialoprotein (BSP), a later osteogenic maker, was detected for MG-63 on TCP, in both C and OS media (Fig 13a). MG-63 C on BCP had increased expression of RUNX2 on days 7 and 14 (Fig. 13b). On day 7, the expression of SOX9 was higher for MG-63 C on BCP than for MG-63 OS on BCP (Fig. 13b). In OS media, MG-63 on BCP showed increased co-expression of RUNX2 and SOX9 on day 14. Pre-osteoblasts remarked expressed BSP (bone sialoprotein) only when cultured on BCP, in both media, on day 14. Here, BSP expression tended to be higher in C media compared to that in OS media (Fig. 13b).

**Fig. 14:**
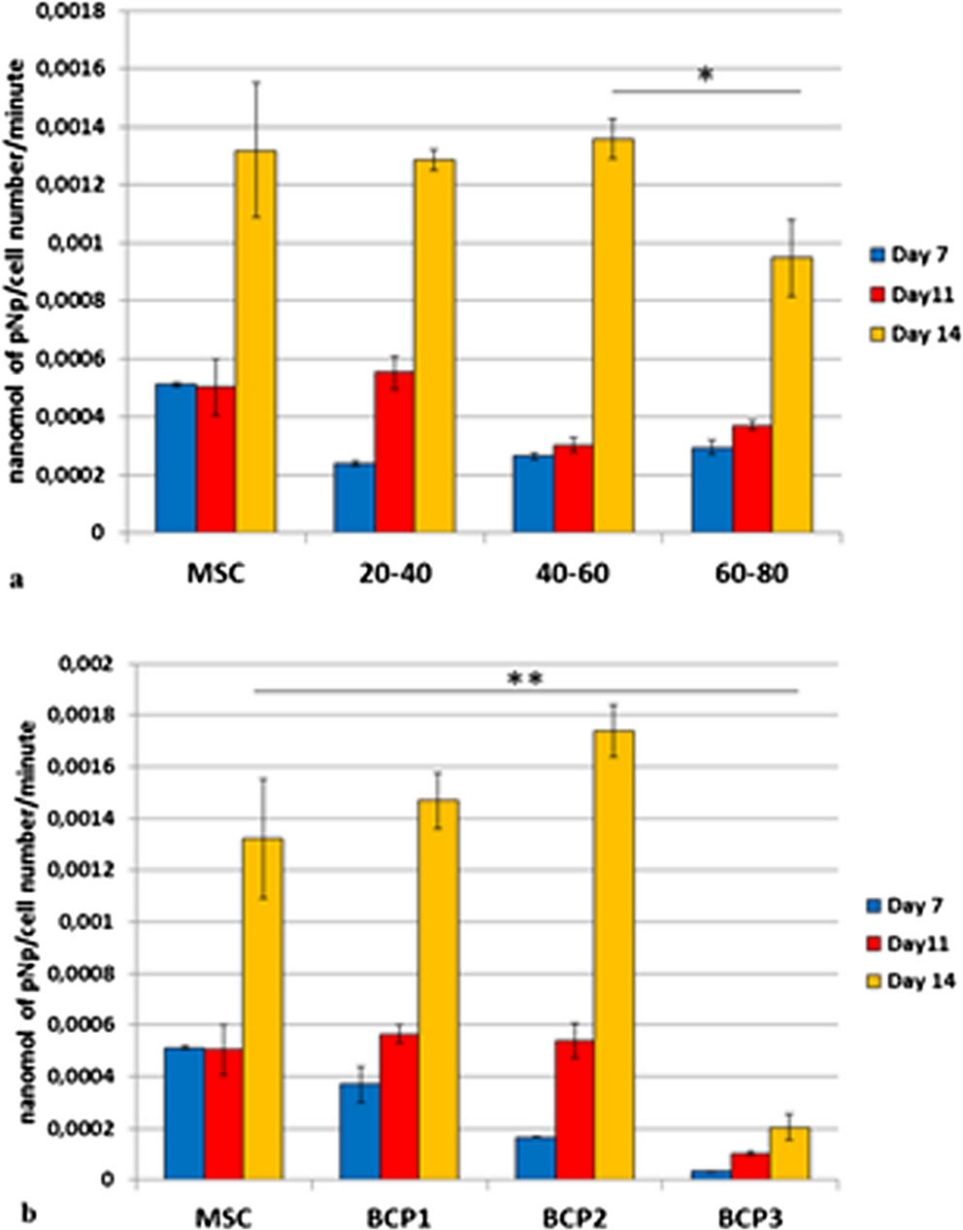
Alkaline phosphatase (ALP) activity of mesenchymal stem cells on the granules (**a**) and blocks (**b**), in osteogenic media. The production of ALP increased over time, from day 1 to 14, for all the groups (*p < 0.05). Cells grown in control medium, with and without biomaterial, did not show ALP activity (data not shown). All groups presented the highest production of ALP at day 14. BCP2 at that time point (day 14) showed the highest ALP activity among all groups (**p < 0.05). MSC, G20-40 and G40-60 were statistically equivalent but were higher than G60-80 at day 14 (*p < 0.05). Values are mean ± SE

The alkaline phosphatase (ALP) activity of MSCs on polystyrene plate and on the six BCPs in OS media increased over time and reached the highest values at day 14 (Fig. 14). MSC seeded on bioceramics, granules and blocks, and on tissue culture polystyrene and grown in control media did not show ALP activity (data not shown). Cells on BCP3 in OS medium had the lowest ALP activity and cells on BCP2 in OS medium presented the highest activity amongst all the groups at day 14 (Fig. 14b).

## Discussion

This study compares the effect of six different biphasic calcium phosphate scaffolds, divided into two major groups, granules and blocks, on adhesion, viability, proliferation and osteogenic differentiation of MSCs. It also compares the expression of early markers of osteogenic differentiation of stem cells isolated from distinct sources cultured on a granular form of BCP. Cells are grown in two different culture media, basal (control) and osteoinductive.

Besides differing in physical form (granules of various sizes versus blocks), the BCPs also differed in chemical composition. Variations in both parameters (chemical and physical) have been related to the creation of distinct microenvironments, which can directly modulate cell responses as cells respond to both intrinsic and extrinsic (host microenvironment) factors (Ohgushi and Caplan 1999; Batouli et al. 2003; Rochet et al. 2003; Knothe Tate et al. 2008; Lee et al. 2013).

The biomaterials’ physical structures, including the frequency, size and shape of the pores, are a result of the manufacturing process and have been shown to play a role in several stages of new bone formation, such as cell adhesion and tissue ingrowth, after biomaterial implantation (Cheng et al. 2010; Xia et al. 2014). Although porosity was not quantified in this study, these materials contained pore structures at both the micro- and macroscale. Increased porosity leads to increased specific surface area, which also influences the scaffold solubilization (increased surface area favors solubilization/bioresorption) (Cheng et al. 2010). On the other hand, higher porosity generally lowers biomaterials’ mechanical properties regardless of their chemical composition (Lin et al. 2007). Specific surface area has also been correlated to cell number: the higher the specific surface area (which is the case of BCP surfaces compared to the flat surfaces of tissue culture plates), the higher the number of attached cells (O’Brien et al. 2005). Scaffolds’ solubilization/bioresorption are a function not only of physical structure but also of the scaffold’s chemical composition and crystallinity. Amorphous biomaterials, such as tricalcium phosphates, favor solubilization over the crystalline ones, such as hydroxyapatites (LeGeros 1993; Lee et al. 2013).

Whereas macroporosity favors cell colonization and tissue ingrowth, the microporosity has been related mostly to the biomaterial’s capacity to adsorb biological fluids (including the entrapment of proteins and other molecules—exogenous or those presented in the site of surgical implantation) (Ripamonti 1996; Cheng et al. 2010; Schumacher et al. 2010; Verron et al. 2010; Yuan et al. 2010). Such protein adsorption, which is also a consequence of the biomaterials’ surface charge and wettability, has been considered the major leading factor responsible for the intrinsic osteoinductivity elicited by some but not all BCPs (Ripamonti 1996; Cheng et al. 2010; Yuan et al. 2010). Considering that the BCP scaffolds analyzed here were rich in micropores, this may have been a contributing factor for the upregulation of osteopontin and bone sialoprotein, two late markers of osteogenic differentiation, observed with cells grown in control media. The nanostructured surface may also have contributed to such response as already reported in the literature (Li et al. 2012). These findings suggested that the BCPs may be osteoinductive and not only osteoconductive materials.

Here, the influence of presentation form on cell response was made evident mainly by the comparison of the granules with the BCP1 block. Overall granules favored cell proliferation and differentiation over blocks. In control medium, larger granules promoted cell differentiation, as observed by gene expression analyses, compared to smaller ones. Moreover, the presence of physical features, specifically micro- and macropores of a biomaterial can substantially improve cell viability, proliferation and differentiation as observed with cells on BCP2 group. The importance of chemical composition has been emphasized by the comparison of BCP1 and BCP3 groups. Calcium phosphate bioceramics have been shown to enrich the microenvironment through calcium and phosphate ion release (Lin et al. 2007; Lee et al. 2013; Shih et al. 2014), otherwise provided by the calcium levels in the media (Huang et al. 2007) and the addition of β-glycerophosphate (a source of phosphate ions) in the osteogenic induction media (Beck et al. 2000). An optimal balance of calcium and phosphate ions in the microenvironment should exist in order to promote a favorable cell response. However, there is evidence in the literature demonstrating that the concentrations of the released ions from calcium phosphate-based biomaterials—hydroxyapatite, hydroxyapatite-collagen composite and calcium metaphosphate—do not interfere in adhesion and proliferation of adipose-derived stem cells and osteoblast-like cells (MC3T3) (Lee et al. 2013). In spite of that, hydroxyapatites have elicited greater expression of alkaline phosphatase m-RNA than hydroxyapatite-collagen composite and calcium metaphosphate (Lee et al. 2013).

Cell proliferation and differentiation are highly coordinated and inversely correlated processes: the more undifferentiated the cells, the higher their proliferative potential (Zhu and Skoultchi 2001). An increase in the number of cells cultured in osteogenic media at day 11 may indicate an efficient MSC proliferation prior to osteogenic differentiation, which occurred at day 14. Such an event has been previously reported and related to the proliferative stage that is part of the osteogenic differentiation (Huang et al. 2007). In our study, cells cultured in OS media were lower in number compared to those in control media.

In the present study, the alkaline phosphatase activity remained low in the groups where cells were cultured in control media, with and without biomaterials. These data suggest that the bioceramics may have induced the osteogenic differentiation of MSCs (observed by the gene expression analyses) but cells may not have progressed within the osteoblastic lineage maturation. Although the ALP measurement has been commonly used to demonstrate osteogenic differentiation, this assay should not be considered conclusive (Weir and Xu 2010).

However, for a biomaterial to be osteoinductive, it must trigger (at early stages of differentiation) genes responsible for directing stem cell fate. Osteoinduction, i.e., the commitment of undifferentiated and/or immature cells toward the osteoprogenitor cell lineage, represents the starting point of the highly coordinated process of osteogenesis (Albrektsson and Johansson 2001; Baglio et al. 2013). In order to test the osteoinductive capacity of the BCP, we investigated the expression of genes responsible for the determination of stem cell fate, at early stages of differentiation (RUNX2, SOX9, PPARγ), of multiple cell types. Therefore, stem cells isolated from different sources (bone marrow, adipose tissue and dental pulp) and pre-osteoblasts (MG-63) were cultured on the BCP that showed the highest expression of BSP and OPN, i.e., G20-40.

The levels and patterns of gene expression observed with the stem cells cultured directly on TCP and in C media demonstrated that they are inherently distinct. Variations in the yield of cells, colony frequency, multipotency, proliferation rate, immunomodulatory properties, cytokine secretion, morphology, immunophenotype, transcriptome and proteome are some of the differences that have been previously reported (Shi et al. 2001; Kern et al. 2006; Bochev et al. 2008; Peng et al. 2008; Rebelatto et al. 2008; Huang et al. 2009; Strioga et al. 2012; Hu et al. 2013; Jin et al. 2013; Melief et al. 2013).

Mesenchymal fate and osteochondrogenesis, for endochondral bone formation, are regulated by intricate pathways where RUNX2 and SOX9 are the key transcription factors (Zou et al. 2006; Grässel et al. 2009; Cheng and Genever 2010). Both are co-expressed by osteochondroprogenitor precursor cells that are found in the adult periosteum and endosteum and in fetal perichondrium, as well as in mandibular secondary cartilages such as in the mandibular condyle and Meckel’s cartilage (Leucht et al. 2008; Zhang et al. 2013).

RUNX2 (aka Osf2/Cbfa1, Pepb2α1 or AML3) is a critical regulator of skeletal development, being observed in the mesenchymal condensations of the developing skeleton, and is the master factor that controls osteoblastic differentiation and chondrocyte maturation (Karsenty 1998; Lian et al. 2006; Zou et al. 2006; Komori 2010; Ding et al. 2012; Nishimura et al. 2012). RUNX2 integrates signaling pathways such as BMP/TGFbeta, Wnt, and Src (Lian et al. 2006), and activates the promoters of several bone protein genes such as COL1a1 (Komori 2010). On the other hand, SOX9 is known to be a marker gene of chondrocytic phenotype besides playing pivotal roles in the organogenesis of several organs (Akiyama et al. 2002; Akiyama 2008; Nishimura et al. 2012; Zhang et al. 2013). The co-expression of RUNX2 and SOX9 resembles the pattern of gene expression normally observed in endochondral bone formation (Leucht et al. 2008; Cheng et al. 2010).

PPARγ is the master regulator of adipogenesis (Lee et al. 2013). Its suppression has been shown to increase the mRNA levels of BMP2, RUNX2, osteocalcin and alkaline phosphatase of ADSC, thus enhancing its osteogenic differentiation (Lee et al. 2013). The maintenance of PPARγ expression by ADSC observed throughout this study suggests that long-term analysis of in vivo bone formation should be performed to verify the quality of the newly formed bone and its capacity to reestablish the physiological turn-over of the bone tissue. Conversely, ADSCs have been shown to be more promising to reconstitute hematopoiesis of irradiated mice than MSCs (Nakao et al. 2010).

DPSC expressed predominantly RUNX2 and SOX9. However, it is important to note that odontoblast and osteoblast share potent regulators and are similar in terms of immunophenotype and the expression of matrix proteins (Nakashima et al. 1994; Gronthos et al. 2000). Nevertheless, the application of DPSC for bone tissue engineering strategies is questioned due to evidence that these cells generate a dentin-like structure in vivo instead of lamellar bone as observed with MSCs (Gronthos et al. 2000). On the other hand, other authors have reported the formation of mature bone using dental pulp stem cells isolated from deciduous teeth instead of permanent teeth (de Mendonça Costa et al. 2008).

Bone sialoprotein (BSP) encodes a major bone matrix structural phosphoprotein that presents an Arg-Gly-Asp (RGD) cell-attachment sequence (Oldberg et al. 1988) and is known to be involved in the nucleation of hydroxyapatite during bone mineralization (Ogata 2008). The fact that pre-osteoblasts expressed BSP only when cultured on BCP scaffolds suggests that these biomaterials may foster the progression of pre-osteoblast differentiation toward a more mature cell phase than TCP. SOX2 is an important transcription factor for the maintenance of the self-renewal capacity of embryonic stem cells, among other functions (Avilion et al. 2003; Luo et al. 2013). It was used as a negative control in this study. SOX2 was detected at very low levels for MSCs but not for all other cell types.

The significance of this study relies on the demonstration that bioceramics with either similar chemical composition, but distinct physical features or vice versa, elicit diverse response (proliferation vs. differentiation) of stem cells and that these, when isolated from various tissues, differ in their gene expression profile at early stages of osteogenic differentiation when in contact with the same type of scaffold (equal chemical and physical structure). These data may be taken into account for further tissue engineering strategies.

## Conclusion

This study concluded that stem cells isolated from various origins are inherently different and their behavior can be influenced by BCP scaffolds. Bioceramics, depending on their chemical composition and physical features, may favor either stem cell proliferation (small granules) or differentiation (large granules). Blocks of bioceramics presenting rough surfaces and a higher number of micro- and macroporosity favor cell viability, proliferation and differentiation over biomaterials with smooth surfaces and less porosity, even if their chemical composition is considered less favorable for osteogenesis. Long-term in vivo studies will be required to determine the quality of newly formed bone promoted by stem cells from different tissue sources.

## Abbreviations

ADSC: Adipose-derived stem cell
ALP: Alkaline phosphatase
BCP: Biphasic calcium phosphate bioceramics
BSP: Bone sialoprotein
β-TCP: Beta tricalcium phosphate
C: Control (basal) medium
CI: Confidence interval
DPSC: Dental pulp stem cell
HA: Hydroxyapatite
MSC: Mesenchymal stem cell
OPN: Osteopontin
OS: Osteoinductive medium
TCP: Tissue culture plate
